# Supplementary material for: Experimental time-reversed adaptive Bell measurement towards all-photonic quantum repeaters
Source: Nat Commun. 2019 Jan 28;10:378. doi: 10.1038/s41467-018-08099-5 (PMC6349889; doi:10.1038/s41467-018-08099-5)
Supplement: Supplementary file 1 — Supplementary Information [file 41467_2018_8099_MOESM1_ESM.pdf]

Supplementary Information—Experimental time-reversed adaptive Bell measurement towards all-photonic quantum repeaters

Yasushi Hasegawa *et al.*

Supplementary Note 1. ACTION OF THE TYPE-II FUSION GATE

Here we clarify the action of the type-II fusion gate. The type-II fusion gate is composed of a PBS oriented at 45°, followed by number-resolving and polarization-resolving detectors on both output modes. Here, the two input spatial modes of the oriented PBS are referred to as mode a and mode b, while its two output spatial modes are referred to as mode c and mode d. Besides, for $x = a, b, c, d$, let $\hat{x}_H$ and $\hat{x}_V$ be annihilation operators of mode x associated with horizontally polarized photons and vertically polarized photons, respectively. Then, the action of the oriented PBS is described by

$$\hat{c}_H = (\hat{a}_H + \hat{a}_V + \hat{b}_H - \hat{b}_V)/2, \tag{1}$$
$$\hat{c}_V = (\hat{a}_H + \hat{a}_V - \hat{b}_H + \hat{b}_V)/2, \tag{2}$$
$$\hat{d}_H = (\hat{a}_H - \hat{a}_V + \hat{b}_H + \hat{b}_V)/2, \tag{3}$$
$$\hat{d}_V = (-\hat{a}_H + \hat{a}_V + \hat{b}_H + \hat{b}_V)/2. \tag{4}$$

Hence, the single-photon detection at the output mode (corresponding to annihilation $\hat{c}_H, \hat{c}_V, \hat{d}_H$ or $\hat{d}_V$) works as the X-basis measurement on mode a or mode b if the modes a and b include a photon in total. On the other hand, the two-photon detection events at the output mode are characterized by

$$\hat{c}_H^2 = \frac{1}{4}(\hat{a}_H^2 + \hat{a}_V^2 + \hat{b}_H^2 + \hat{b}_V^2) + \frac{1}{2}(\hat{a}_H\hat{a}_V - \hat{b}_H\hat{b}_V) + \frac{1}{2}(\hat{a}_H\hat{b}_H + \hat{a}_V\hat{b}_H - \hat{a}_H\hat{b}_V - \hat{a}_V\hat{b}_V), \tag{5}$$
$$\hat{c}_V^2 = \frac{1}{4}(\hat{a}_H^2 + \hat{a}_V^2 + \hat{b}_H^2 + \hat{b}_V^2) + \frac{1}{2}(\hat{a}_H\hat{a}_V - \hat{b}_H\hat{b}_V) + \frac{1}{2}(-\hat{a}_H\hat{b}_H - \hat{a}_V\hat{b}_H + \hat{a}_H\hat{b}_V + \hat{a}_V\hat{b}_V), \tag{6}$$
$$\hat{d}_H^2 = \frac{1}{4}(\hat{a}_H^2 + \hat{a}_V^2 + \hat{b}_H^2 + \hat{b}_V^2) - \frac{1}{2}(\hat{a}_H\hat{a}_V - \hat{b}_H\hat{b}_V) + \frac{1}{2}(\hat{a}_H\hat{b}_H - \hat{a}_V\hat{b}_H + \hat{a}_H\hat{b}_V - \hat{a}_V\hat{b}_V), \tag{7}$$
$$\hat{d}_V^2 = \frac{1}{4}(\hat{a}_H^2 + \hat{a}_V^2 + \hat{b}_H^2 + \hat{b}_V^2) - \frac{1}{2}(\hat{a}_H\hat{a}_V - \hat{b}_H\hat{b}_V) + \frac{1}{2}(-\hat{a}_H\hat{b}_H + \hat{a}_V\hat{b}_H - \hat{a}_H\hat{b}_V + \hat{a}_V\hat{b}_V), \tag{8}$$
$$\hat{c}_H\hat{c}_V = \frac{1}{4}(\hat{a}_H^2 + \hat{a}_V^2 - \hat{b}_H^2 - \hat{b}_V^2) + \frac{1}{2}(\hat{a}_H\hat{a}_V + \hat{b}_H\hat{b}_V), \tag{9}$$
$$\hat{d}_H\hat{d}_V = \frac{1}{4}(-\hat{a}_H^2 - \hat{a}_V^2 + \hat{b}_H^2 + \hat{b}_V^2) + \frac{1}{2}(\hat{a}_H\hat{a}_V + \hat{b}_H\hat{b}_V), \tag{10}$$
$$\hat{c}_H\hat{d}_H = \frac{1}{4}(\hat{a}_H^2 - \hat{a}_V^2 + \hat{b}_H^2 - \hat{b}_V^2) + \frac{1}{2}(\hat{a}_H\hat{b}_H + \hat{a}_V\hat{b}_V), \tag{11}$$
$$\hat{c}_V\hat{d}_V = \frac{1}{4}(-\hat{a}_H^2 + \hat{a}_V^2 - \hat{b}_H^2 + \hat{b}_V^2) + \frac{1}{2}(\hat{a}_H\hat{b}_H + \hat{a}_V\hat{b}_V), \tag{12}$$
$$\hat{c}_V\hat{d}_H = \frac{1}{4}(\hat{a}_H^2 - \hat{a}_V^2 - \hat{b}_H^2 + \hat{b}_V^2) + \frac{1}{2}(\hat{a}_H\hat{b}_V + \hat{a}_V\hat{b}_H), \tag{13}$$
$$\hat{c}_H\hat{d}_V = \frac{1}{4}(-\hat{a}_H^2 + \hat{a}_V^2 + \hat{b}_H^2 - \hat{b}_V^2) + \frac{1}{2}(\hat{a}_H\hat{b}_V + \hat{a}_V\hat{b}_H). \tag{14}$$

Note that each of mode a and mode b includes only a single photon at most. Then, the first terms in the right-hand-side of all these equations and the second terms in the right-hand-side of Supplementary Eqs. (5)-(10) have no meaning. Therefore, the two-photon detection events corresponding to Supplementary Eqs. (5)-(8) work as the X-basis measurement on modes a and b, the two-photon detection events corresponding to Supplementary Eqs. (9) and (10) never occur, and the two-photon detection events corresponding to Supplementary Eqs. (11)-(14) work as the Bell measurement on modes a and b.

Supplementary Note 2. ASYMPTOTIC FINAL SECRET KEY RATE FORMULA

In this Supplementary Note, we present the asymptotic final secret key formula $G := \max_l G_l$ of our protocol defined in our main text, by assuming the use of photon-number-resolving detectors with quantum efficiency $\eta = 1-\epsilon_0$. Using the formula, one can reproduce the key rates in Fig. 6.

We first derive the final form of the GHZ state that is distributed to Alice and Bob when our protocol succeeds. We begin by noting that a type-II fusion gate at the node C in our protocol aims at performing a connection process between an m-partite GHZ state

$$|G_{\rm g}^m\rangle_{x_1x_2\cdots x_m} := \frac{1}{\sqrt{2}}(|HH\cdots H\rangle_{x_1x_2\cdots x_m} + |VV\cdots V\rangle_{x_1x_2\cdots x_m}), \tag{15}$$

and a bipartite GHZ state $|G_{\rm g}^2\rangle_{\alpha_1 a_1}$, by using photon-number-resolving detectors with quantum efficiency η. The bipartite GHZ state $|G_{\rm g}^2\rangle_{\alpha_1 a_1}$ is associated with entanglement prepared by Alice (or Bob) locally, and its half α_1 is to be sent to the node C through a lossy channel. First, suppose that the type-II fusion gate is applied to modes $x_1\alpha_1$. We will use an equivalent description of the type-II fusion gate such that, as long as all photon detectors of a type-II fusion gate have the same quantum efficiency, the description of the type-II fusion gate is equivalent to individual lossy channels on modes $x_1\alpha_1$, followed by the ideal type-II fusion gate.

According to Supplementary Note 1, when the measurement outcome of the type-II fusion gate on modes $x_1\alpha_1$ announces arrival of two photons in total, it works as the Bell or X-basis measurement on modes $x_1\alpha_1$, even if the state $|G_{\rm g}^m\rangle_{x_1x_2\cdots x_m}|G_{\rm g}^2\rangle_{\alpha_1 a_1}$ has undergone lossy channels on $x_1\alpha_1$ before the measurement. Thus, in either case, the remaining state is in a GHZ state, such as $|G_{\rm g}^m\rangle_{a_1x_2\cdots x_m}$ and $|G_{\rm g}^{m-1}\rangle_{x_2\cdots x_m}$ (up to the freedom of Pauli local unitaries). Hence, to derive the form of the final GHZ state for Alice and Bob though a success event of our protocol, without loss of generality, we can assume that all the type-II fusion gates at the node C informing us of arrival of two photons have already been performed and the remaining system is in an n-partite GHZ state $|G_{\rm g}^n\rangle_{x_1x_2\cdots x_n}$ for some n. Then, all the other type-II fusion gates at the node C, which must announce arrival of a single photon in a success event of our protocol, are applied to this remaining system. Therefore, for deriving the form of the final GHZ state between Alice and Bob, it is sufficient to consider only what happens for the state $|G_{\rm g}^n\rangle_{x_1x_2\cdots x_n}$ if we apply the type-II fusion gates informing us of arrival of a single photon. Let us consider this in what follows.

If an n-partite GHZ state $|G_{\rm g}^n\rangle_{x_1x_2\cdots x_n}$ is subjected to a lossy channel with transmittance ζ corresponding to the photon loss for the mode x_1, the state after the loss is described by

$$|\psi_\zeta^n\rangle_{x_1;x_2\cdots x_n;e_1} := \sqrt{\zeta}|G_{\rm g}^n\rangle_{x_1x_2\cdots x_n}|0\rangle_{e_1} + \sqrt{1-\zeta}|0\rangle_{x_1}|G_{\rm g}^n\rangle_{x_2\cdots x_n e_1}, \tag{16}$$

where system e_1 is an environment system and $|0\rangle$ represents the vacuum state. With the above equivalent description of the type-II fusion gate and this state description, we can regard the connection process as the application of the ideal type-II fusion gate to modes $x_1\alpha_1$ in state

$$\begin{aligned}|\psi_\eta^n\rangle_{x_1;x_2\cdots x_n;e_1}|\psi_\xi^2\rangle_{\alpha_1;a_1;E_1} = {}& (\sqrt{\eta}|G_{\rm g}^n\rangle_{x_1x_2\cdots x_n}|0\rangle_{e_1} + \sqrt{1-\eta}|0\rangle_{x_1}|G_{\rm g}^n\rangle_{x_2\cdots x_n e_1}) \\ & \otimes (\sqrt{\xi}|G_{\rm g}^2\rangle_{\alpha_1 a_1}|0\rangle_{E_1} + \sqrt{1-\xi}|0\rangle_{\alpha_1}|G_{\rm g}^2\rangle_{a_1E_1}),\end{aligned} \tag{17}$$

where note that the bipartite GHZ state is also subjected to a lossy channel on the mode α_1, corresponding to the photon loss during the travel to the node C as well as the effect of non-unity quantum efficiency. Suppose that the ideal type-II fusion gate on modes $x_1\alpha_1$ works as, for instance, the application of $(\langle D|_{x_1}\langle 0|_{\alpha_1} + \langle 0|_{x_1}\langle A|_{\alpha_1})/\sqrt{2}$, corresponding to the event of Supplementary Eq. (1). This converts the state $|\psi_\eta^n\rangle_{x_1;x_2\cdots x_n;e_1}|\psi_\xi^2\rangle_{\alpha_1;a_1;E_1}$ into unnormalized state

$$\begin{aligned}|\phi^1\rangle_{x_2\cdots x_n e_1 a_1 E_1} :={}& \frac{1}{\sqrt{2}}(\langle D|_{x_1}\langle 0|_{\alpha_1} + \langle 0|_{x_1}\langle A|_{\alpha_1})|\psi_\eta^n\rangle_{x_1;x_2\cdots x_n;e_1}|\psi_\xi^2\rangle_{\alpha_1;a_1;E_1} \\ ={}& \frac{1}{2}(\sqrt{\eta(1-\xi)}|G_{\rm g}^{n-1}\rangle_{x_2\cdots x_n}|0\rangle_{e_1}|G_{\rm g}^2\rangle_{a_1E_1} + \sqrt{(1-\eta)\xi}|G_{\rm g}^n\rangle_{x_2\cdots x_n e_1}|G_{\rm g}'^1\rangle_{a_1}|0\rangle_{E_1}),\end{aligned} \tag{18}$$

where $|G_{\rm g}'^n\rangle_{x_1x_2\cdots x_n} := \hat{Z}_{x_1}|G_{\rm g}^n\rangle_{x_1x_2\cdots x_n}$ with $\hat{Z}_{x_1} := |H\rangle\langle H|_{x_1} - |V\rangle\langle V|_{x_1}$.

To see a tendency, suppose that there is one more application of a type-II fusion gate informing us of arrival of a single photon to modes $x_2\alpha_2$ in unnormalized state $|\phi^1\rangle_{x_2\cdots x_m e_1 a_1 E_1}|G_{\rm g}^2\rangle_{\alpha_2 a_2}$. Again, we regard this type-II fusion gate as the application of lossy channels on $x_2\alpha_2$, followed by the ideal type-II fusion gate. For the lossy channel on mode x_2, the state in Supplementary Eq. (18) evolves into

$$|\phi_\eta^1\rangle_{x_2\cdots x_n e_2 e_1 a_1 E_1} := \frac{1}{2}(\sqrt{\eta(1-\xi)}|\psi_\eta^{n-1}\rangle_{x_2;x_3\cdots x_n;e_2}|0\rangle_{e_1}|G_{\rm g}^2\rangle_{a_1E_1} + \sqrt{(1-\eta)\xi}|\psi_\eta^n\rangle_{x_2;e_1x_3\cdots x_n;e_2}|G_{\rm g}'^1\rangle_{a_1}|0\rangle_{E_1}). \tag{19}$$

Then, the ideal type-II fusion gate on modes $x_2\alpha_2$ works as, again for instance, the application of $(\langle D|_{x_2}\langle 0|_{\alpha_2}+\langle 0|_{x_2}\langle A|_{\alpha_2})/\sqrt{2}$, corresponding to the event of Supplementary Eq. (1). This converts the state $|\phi^1_\eta\rangle_{x_2\cdots x_n e_2 e_1 a_1 E_1}|\psi^2_\xi\rangle_{\alpha_2;a_2;E_2}$ into unnormalized state

$$\begin{aligned}
|\phi^2\rangle_{x_3\cdots x_n e_2 a_2 E_2 e_1 a_1 E_1} :=& \frac{1}{\sqrt{2}}(\langle D|_{x_2}\langle 0|_{\alpha_2}+\langle 0|_{x_2}\langle A|_{\alpha_2})|\phi^1_\eta\rangle_{x_2\cdots x_n e_2 e_1 a_1 E_1}|\psi^2_\xi\rangle_{\alpha_2;a_2;E_2}\\
=& \frac{1}{2}\Bigg\{\sqrt{\eta(1-\xi)}\left[\frac{1}{\sqrt{2}}(\langle D|_{x_2}\langle 0|_{\alpha_2}+\langle 0|_{x_2}\langle A|_{\alpha_2})|\psi^{n-1}_\eta\rangle_{x_2;x_3\cdots x_n;e_2}|\psi^2_\xi\rangle_{\alpha_2;a_2;E_2}\right]|0\rangle_{e_1}|G^2_{\rm g}\rangle_{a_1E_1}\\
&+\sqrt{(1-\eta)\xi}\left[\frac{1}{\sqrt{2}}(\langle D|_{x_2}\langle 0|_{\alpha_2}+\langle 0|_{x_2}\langle A|_{\alpha_2})|\psi^{n}_\eta\rangle_{x_2;e_1x_3\cdots x_n;e_2}|\psi^2_\xi\rangle_{\alpha_2;a_2;E_2}\right]|0\rangle_{E_1}|G'^1_{\rm g}\rangle_{a_1}\Bigg\}\\
=& \frac{1}{4}(\sqrt{\eta^2(1-\xi)^2}|G^{n-2}_{\rm g}\rangle_{x_3\cdots x_n}|0\rangle_{e_2}|G^2_{\rm g}\rangle_{a_2E_2}|0\rangle_{e_1}|G^2_{\rm g}\rangle_{a_1E_1}+\sqrt{\eta(1-\eta)\xi(1-\xi)}|G^{n-1}_{\rm g}\rangle_{x_3\cdots x_ne_2}|G'^1_{\rm g}\rangle_{a_2}|0\rangle_{E_2}|0\rangle_{e_1}|G^2_{\rm g}\rangle_{a_1E_1}\\
&+\sqrt{\eta(1-\eta)\xi(1-\xi)}|G^{n-1}_{\rm g}\rangle_{e_1x_3\cdots x_n}|0\rangle_{e_2}|G^2_{\rm g}\rangle_{a_2E_2}|G'^1_{\rm g}\rangle_{a_1}|0\rangle_{E_1}+\sqrt{(1-\eta)^2\xi^2}|G^{n}_{\rm g}\rangle_{e_1x_3\cdots x_ne_2}|G'^1_{\rm g}\rangle_{a_2}|0\rangle_{E_2}|G'^1_{\rm g}\rangle_{a_1}|0\rangle_{E_1}),
\end{aligned} \tag{20}$$

where we used Supplementary Eq. (18).

Therefore, from Supplementary Eqs. (18) and (20), if there are k type-II fusion gates which announce arrival of a single photon, up to the freedom of local Pauli unitaries, the final GHZ state will be described by

$$\hat{\rho}^{(k)}_{x_{k+1}\cdots x_n}=F_k|G^{n-k}_{\rm g}\rangle\langle G^{n-k}_{\rm g}|_{x_{k+1}\cdots x_n}+(1-F_k)\frac{1}{2}(|HH\cdots H\rangle\langle HH\cdots H|_{x_{k+1}\cdots x_n}+|VV\cdots V\rangle\langle VV\cdots V|_{x_{k+1}\cdots x_n}), \tag{21}$$

where

$$F_k=\frac{\eta^k(1-\xi)^k}{[\eta(1-\xi)+(1-\eta)\xi]^k}. \tag{22}$$

In a success event of our protocol, a part of system $x_{k+1}\cdots x_n$ is held by Alice and the other part is held by Bob. Then, if Alice and Bob distil the key from the GHZ state by performing the Z-basis measurement, they observe that the bit error rate e^Z_k and the phase error rate e^X_k are

$$e^Z_k=0, \tag{23}$$

$$e^X_k=\frac{1}{2}(1-F_k). \tag{24}$$

Thus, in this case, their asymptotic key rate is described by $1-h(e^Z_k)-h(e^X_k)$, where $h(x):=-x\log_2 x-(1-x)\log_2(1-x)$.

Let us move on to the derivation of the success probabilities. Suppose that there are $k_{\rm L}$ left-hand-side ($k_{\rm R}$ right-hand-side) type-II fusion gates at the node C which have been applied to a pulse from Alice (from Bob) and a locally prepared qubit and which have announced arrival of a single photon. The success condition of our protocol requires all the other $l-k_{\rm L}$ left-hand-side ($l-k_{\rm R}$ right-hand-side) type-II fusion gates to have observed arrival of two photons, among which there must be, at least, a type-II fusion gate on having worked as the Bell measurement. Hence, the probability $R(k_{\rm L},k_{\rm R})$ with which our protocol succeeds for given $k_{\rm L}(\le l-1)$ and $k_{\rm R}(\le l-1)$ is

$$\begin{aligned}
R(k_{\rm L},k_{\rm R})=&\binom{l}{k_{\rm L}}[\eta(1-\xi)+(1-\eta)\xi]^{k_{\rm L}}(\eta\xi)^{l-k_{\rm L}}\left[1-\left(\frac{1}{2}\right)^{l-k_{\rm L}}\right]\\
&\times\binom{l}{k_{\rm R}}[\eta(1-\xi)+(1-\eta)\xi]^{k_{\rm R}}(\eta\xi)^{l-k_{\rm R}}\left[1-\left(\frac{1}{2}\right)^{l-k_{\rm R}}\right],
\end{aligned} \tag{25}$$

where we used the fact that a type-II fusion gate works as the Bell measurement with probability 1/2 when it receives two photons.

Therefore, the asymptotic average secret bits G_l of our protocol per the number l of multiplexing is

$$G_l=\frac{1}{l}\sum_{k_{\rm L},k_{\rm R}=0}^{l-1}R(k_{\rm L},k_{\rm R})(1-h(e^Z_{k_{\rm L}+k_{\rm R}})-h(e^X_{k_{\rm L}+k_{\rm R}})), \tag{26}$$

Supplementary Table I: Examples of the optimal numbers $l_{\rm opt}$ of multiplexing.

Quantum efficiency η	$L = 100$ km	$L = 200$ km	$L = 400$ km	$L = 800$ km
1	$l_{\rm opt} = 24$	$l_{\rm opt} = 236$	$l_{\rm opt} = 22299$	$l_{\rm opt} = 157504623$
0.99999	$l_{\rm opt} = 24$	$l_{\rm opt} = 235$	$l_{\rm opt} = 14546$	$l_{\rm opt} = 49984$
0.9999	$l_{\rm opt} = 24$	$l_{\rm opt} = 222$	$l_{\rm opt} = 8876$	$l_{\rm opt} = 5000$
0.999	$l_{\rm opt} = 22$	$l_{\rm opt} = 149$	$l_{\rm opt} = 486$	$l_{\rm opt} = 500$
0.99	$l_{\rm opt} = 14$	$l_{\rm opt} = 39$	$l_{\rm opt} = 50$	$l_{\rm opt} = 50$
0.9	$l_{\rm opt} = 3$	$l_{\rm opt} = 5$	$l_{\rm opt} = 5$	$l_{\rm opt} = 5$

and the final key rate formula G is

$$G = \max_{l \geq 1} G_l. \tag{27}$$

These key rates can be estimated by using Supplementary Eqs. (22)-(25).

To derive Fig. 6, for the distance L between Alice and Bob, η and ξ are defined as

$$\eta = 1 - \epsilon_0, \tag{28}$$

$$\xi = \eta_{L/2}\eta = e^{-L/(2L_{\rm att})}(1 - \epsilon_0), \tag{29}$$

by using parameter ϵ_0 and attenuation length $L_{\rm att}$ in the main text. Examples of the optimal choice $l_{\rm opt}$ of the number l of multiplexing which maximizes G_l in Supplementary Eq. (27) are shown in Supplementary Table I for given distance L and quantum efficiency η.
